# Generosity Pays in the Presence of Direct Reciprocity: A Comprehensive Study of 2×2 Repeated Games

**DOI:** 10.1371/journal.pone.0035135

**Published:** 2012-04-18

**Authors:** Luis A. Martinez-Vaquero, José A. Cuesta, Angel Sánchez

**Affiliations:** 1 Grupo Interdisciplinar de Sistemas Complejos (GISC), Departamento de Matemáticas, Universidad Carlos III de Madrid, Leganés, Madrid, Spain; 2 Instituto de Biocomputación y Física de Sistemas Complejos (BIFI), Universidad de Zaragoza, Zaragoza, Spain; University of Zaragoza, Spain

## Abstract

By applying a technique previously developed to study ecosystem assembly [Capitán *et al.*, Phys. Rev. Lett. **103**, 168101 (2009)] we study the evolutionary stable strategies of iterated 2
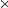
2 games. We focus on memory-one strategies, whose probability to play a given action depends on the actions of both players in the previous time step. We find the asymptotically stable populations resulting from all possible invasions of any known stable population. The results of this invasion process are interpreted as transitions between different populations that occur with a certain probability. Thus the whole process can be described as a Markov chain whose states are the different stable populations. With this approach we are able to study the whole space of symmetric 2
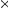
2 games, characterizing the most probable results of evolution for the different classes of games. Our analysis includes quasi-stationary mixed equilibria that are relevant as very long-lived metastable states and is compared to the predictions of a fixation probability analysis. We confirm earlier results on the success of the Pavlov strategy in a wide range of parameters for the iterated Prisoner's Dilemma, but find that as the temptation to defect grows there are many other possible successful strategies. Other regions of the diagram reflect the equilibria structure of the underlying one-shot game, albeit often some non-expected strategies arise as well. We thus provide a thorough analysis of iterated 2
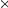
2 games from which we are able to extract some general conclusions. Our most relevant finding is that a great deal of the payoff parameter range can still be understood by focusing on win-stay, lose-shift strategies, and that very ambitious ones, aspiring to obtaining always a high payoff, are never evolutionary stable.

## Introduction

Cooperation has been reported at practically every level of biological organization [1] and, in fact, it has been argued to play a key role in the major steps of evolution [2]. In spite of its widespread presence, cooperation faces a central problem, namely the vulnerability of cooperators to being exploited by selfish partners, as realized already by Darwin [3]. The need for a sophisticated, subtle explanation of cooperation was recognized early on by Hamilton [4], [5] and Trivers [6], who based their theories of cooperation on genetic relatedness (kin selection) and on the logic of repeated interactions (reciprocal altruism or direct reciprocity), respectively. Subsequently, other theories have been put forward as possible explanations for the appearance and emergence of cooperation [7] with different degrees of success and applicability.

Among the theories of cooperation, direct reciprocity has received a lot of attention, in particular from the theoretical viewpoint. The reason for this is twofold: on the one hand, as Dugatkin [8] puts it, reciprocity is a type of cooperation that is far from trivial to explain and, being such a hard challenge, it requires more work. On the other hand, albeit rare in most other social animals [9] including primates [10], [11], reciprocity is one of the most important forms of human cooperation [1], [12], probably since almost two million years ago [13], [14], as reciprocity appears to be an unavoidable consequence of small group size, given the cognitive abilities of humans [15]. Thus, direct reciprocity has been studied by many authors, starting from the original proposal by Trivers [6], relevant contributions including Refs. [16]–[25]. Practically all these works deal with the Prisoner's Dilemma [26] as the paradigm through which the discussion takes place (for a recent summary, see chapter 3 in [27]).

The large amount of research done on the iterated Prisoner's Dilemma has allowed to reach some important conclusions. Thus, the famous computer tournaments organized by Axelrod [12] showed that a simple strategy, tit-for-tat (TFT), in which players started cooperating and then repeated the opponent's previous action, was the most successful among those submitted to play the iterated Prisoner's Dilemma. Subsequent works pointed out the relevance of a less vengeful version, generous TFT [28] and, furthermore, that TFT could be outperformed by Pavlov, a win-stay, lose-shift type of strategy [20]. In order to systematize these findings, it was proposed [29], [30] to consider finite automata as players. This approach was later improved upon by including noise (errors in performing an action or in perceiving the opponent's action) [31], [32]. The basic idea behind those studies is to consider all possible sets of strategies defined in terms of their action following all possibilities of actions by the focus player and her opponent (memory-one strategies). Noise is included by allowing errors in the implementation of the action with a small probability. Then, starting from a given initial population, strategies face each other and reproduce according to their performance, possibly with mutation. With this dynamics, complicated (chaotic) trajectories as well as cycles involving a number of strategies–such as AllD (always defect), GRIM (always defect after the first defection by the opponent), TFT, Pavlov, and others–can be observed. However, as Sigmund [27] puts it, it is hard to sort out, among the many possible strategies, which one would be selected by evolution; indeed, individual based simulations display particular contingencies and few robust predictions. On the other hand, other games can be used as paradigms for the emergence of cooperation and, in fact, Nowak et al. [32] considered the Hawk-Dove or Snowdrift game [33], showing that Pavlov played an important role in it as well.

In this paper, we elaborate on the above discussed issues by benefiting from a completely different approach, recently developed in the context of the emergence and robustness of ecosystems [34]–[36]. Roughly speaking, the idea is to look at the stability of populations of strategies by attempting to invade them with all the other available ones, and then repeat the procedure with the resulting stable populations, and so on and so forth until one has identified all the stable “ecosystems", i.e., all the stable composition of populations. With this procedure, one can compute the probabilities of transition between those stable populations and treat the system as a Markov chain whose states are the stable populations. Subsequently, the theory of Markov chains allows one to identify what are the absorbing and/or recurrent sets and hence the relevant population compositions. The advantage of this method is that it systematically explores all possible compositions instead of relying on random mutations to drive the evolution of the system toward the evolutionary relevant ones. In addition, by means of this technique, we have been able to explore not only the Prisoner's Dilemma game but all the possible symmetric 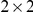 games, thus widening enormously our knowledge of the effects of direct reciprocity on the different forms of possible human interactions and social dilemmas [37].

Our results and conclusions will be presented according to the following scheme. In ‘Methods’ we introduce our model and detail our approach to the problem, describing in depth the procedure we are adapting from studies of ecosystem assembly and also an alternative analysis using fixation probabilities. We also explain how to implement the approach in practical terms by using an acceleration procedure, and the manner in which the relevant results are obtained in terms of the parameters characterizing them, the most important one being the structure of the invasion graph in terms of absorbing and recurrent sets. Our results are collected and explained in the ‘Results’ section, where we analyze all the parameter space of symmetric 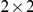 games, identify the strategies appearing in the relevant equilibria and discuss the evolutionary reasons for the composition of those equilibria. Finally, section ‘Discussion’ concludes the paper by summarizing our main results and their implications.

## Methods

### Model

We will consider well-mixed populations in which individuals interact in pairs with a randomly chosen opponent. Whenever two players engage in one such interaction they play an iterated two-strategy game. Each round of this game players can choose among two possible actions, that we generically term C (for cooperate) and D (for defect). If a player plays C she receives 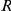 if the opponent also plays C and 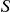 if the opponent plays D; if she plays D instead, she receives 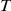 if the opponent plays C and 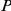 if the opponent also plays D. The payoff obtained by each player is added to her accumulated payoff so far, and a new round of the game takes place with probability 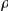.

The game thus described will last exactly 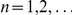 rounds with probability 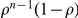, so the expected number of rounds is 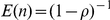. On the other hand, if 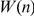 is the payoff collected by a given player in round 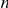, the expected payoff will be
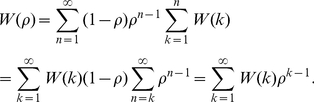
(1)Payoffs are to be compared with each other, so that rescaling them all by the same factor is immaterial. Thus it is convenient to introduce the expected averaged payoff dividing by 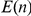, i.e.,
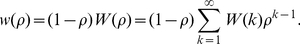
(2)With this definition we can even study the limit 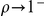, for then, applying a theorem of Frobenius [27],
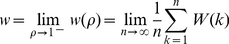
(3)coincides with the average payoff of an infinitively long run of iterations. For simplicity we will henceforth only consider this limit.

Without loss of generality we can set one of the entries of the 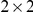 payoff matrix to 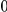 (setting the origin of payoffs) and another one to 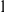 (scaling payoffs by one of them). A common normalization is 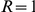 and 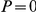 [28], [38]. With this choice 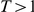 becomes a temptation to defect and 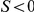 involves a risk in cooperating. With the combination of this two tensions we can parametrize different social dilemmas represented by the Harmony game (no tensions), the Stag Hunt game (risk in cooperating), the Snowdrift or Hawk-Dove game (temptation to defect), or the Prisoner's Dilemma (both tensions).

### Strategies

The available set of strategies for a player involved in an iterated game is virtually infinite, so for practical purposes we must impose strong constraints to the players' behavior that select a finite–hopefully small–number of strategies out of this set. In modeling direct reciprocity memory is an important ingredient, so we will focus on strategies that take into account the past history of the iterated game against the same opponent. It is reasonable to assume that players have a limited ability to remember past actions, so we will focus on strategies that depend only on a fixed number of past rounds. Among them, the simplest and most studied are memory-one strategies [27]. These are the only ones we will be dealing with in this article.

Memory-one strategies are characterized by four parameters, namely the four probabilities of cooperating in the current round, given that the focal player played X and the opponent played Y in the previous round (X,Y
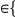
C,D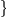). We will denote this probabilities 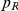, 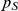, 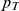, and 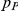, where the subscripts denote the payoffs when the pair XY is CC (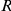), CD (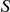), DC (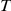), and DD (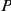). Each of these probabilities is in the interval 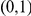, excluding both zero and one because some strategies having these extreme probabilities (e.g., TFT) are unstable against errors [20], [27], [32].

Memory-one strategies with error still form an infinite set of strategies–namely the hypercube 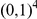. Simulations, though, indicate that interactions are dominated by extreme strategies [20], i.e., those for which probabilities are either 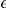 or 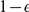, with 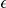 denoting the probability of making a mistake in choosing the action (with notable exceptions like generous TFT). Accordingly we will limit our study to only this kind of strategies, taking 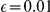. The results did not show qualitative differences when higher values of 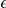 were taken. Thus we are left with a set of 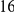 strategies, each characterized by a binary vector 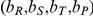 in which 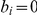 if 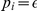 and 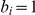 if 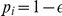.

### Payoff matrix

In order to obtain the 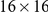 payoff matrix of this game we need to determine the stationary probability with which the four states (CC, CD, DC, and DD) occur. The interaction between two players forms a four-state Markov chain whose transition probability matrix–denoted 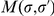
–is given by [27]

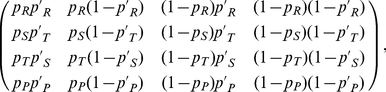
(4)where 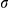 and 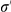 are shorthands for the four-number codes of the strategies of the focal player and her opponent, respectively–whose probabilities are distinguished with a prime. The steady state is described by the left eigenvector 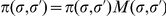, in terms of whose components the average payoff of the focal player when confronted against her opponent will be
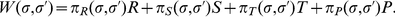
(5)This defines the element 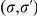 of the payoff matrix.

### Invasion dynamics

We will consider that players may spontaneously change their strategies and adopt any other one. These “mutations"–especially if they occur often–are a source of heterogeneity in populations. In the limit when this mutation rate is very small any change of strategy can be regarded as an attempt of a new strategy to invade a resident population. Given that there are only 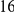 different strategies such invasion attempts can be systematically studied using a method recently introduced in the context of ecological community assembly [34]–[36].

The idea is to construct an invasion graph as follows. We start off from 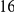 homogeneous populations, each with a different strategy. Every one of these initial states is represented with a node of the invasion graph. Now we invade each of these homogeneous populations with everyone of the 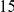 remaining strategies (invasions are assumed to occur at a very small fraction of the total population). The dynamics leads the system to one of the following states: (a) back to the original homogeneous population, (b) to a homogeneous population of the mutant strategy, or (c) to a mixed equilibrium of both strategies. In case (a) nothing is added to the invasion graph; in case (b) a directed link is established from the node corresponding to the original population to the node corresponding to the final population (the link is labeled with the invading strategy); in case (c) a new node is added to the graph representing the new mixed equilibrium and a labeled, directed link goes from the original population to this new node. When every one of the original 16 nodes has been invaded with every one of the other 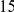 strategies, we focus in the added nodes (mixed equilibria) and try to invade them with each of the remaining 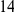 other strategies. Again links are created from these nodes to the nodes the invasion leads to, and new nodes are added to the graph for every new equilibrium found after the invasion. We proceed in this way until no new nodes can be added through invasions.

We have not specified the population dynamics yet. In principle, given the payoff matrix (5), an imitation dynamics can be implemented through the replicator equation [39] under the assumption that populations are infinitely large. Nowak et al. [32] analyzed the replicator dynamics for this kind of strategies. The results show that, as the number of strategies in the population increase, the probability that the population gets engaged in a cycle or a strange attractor increases as well. Our own calculations confirm this fact. The problem with cyclic dynamics is that orbits are structurally unstable, and their very existence is a direct consequence of the infinite population limit under which the replicator dynamics makes sense. Sampling noise introduced by small populations destroys the orbits.

For the above reason, we have chosen a discrete imitation dynamic in a large (albeit finite) population, which we have set to 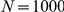 individuals. According to this dynamics two individuals are selected at random from the population; if 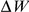 denotes the excess payoff obtained by the opponent compared to that of the focal players, then the latter replaces her strategy by that of the former with probability
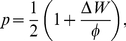
(6)

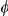
 being the largest possible payoff difference. This probability is 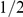 if there is no payoff difference (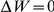, corresponding to random drift) and larger (smaller) than 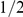 if 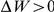 (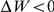). There are other alternative stochastic dynamics that can be implemented [38] and the results may depend on this specific choice. The main reason to choose this one is that it becomes equivalent to the replicator dynamics in the limit 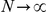. However, even a proportional update like this one could have been implemented in a different way, for instance setting 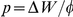 if 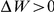 and 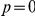 otherwise. The reason to prefer (6) to the latter is that for 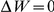 it correctly captures random drift, and thus complies better with the behavior one observes in real systems.

### Fixation processes

A discrete dynamics like the one we are considering here always leads to an asymptotically homogeneous population. Since only mutations (invasions) can introduce new strategies, a homogeneous population is always an absorbing state. Nevertheless, we are going to consider mixed equilibria as well. The rationale for this is that these (unarguably) metastable states have an absorption time into a homogeneous population that grows exponentially with 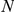, so that the absorbing states becomes virtually unreachable. The existence of these states can be rigorously formalized using the quasi-stationarity concept of Markov chains [40]–although we will not follow such a rigorous approach here, but content ourselves with a practical implementation of it focusing on non-absorbed realizations of the process.

To support our argument, let us calculate the probabilities of the different invasions as fixation probabilities, i.e. the probability that a single invader will eventually be imitated by all the rest of individuals, who play the resident strategy. This probability is given by [27], [41]

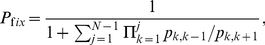
(7)where 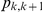 is the probability that an individual of the resident strategy imitates a mutant one (*birth*) and 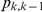 is the probability that an individual of the mutant strategy imitates a resident one (*death*). These probabilities are obtained from the imitation probability (6).

An easy to obtain lower bound to the mean time to fixation is [41]

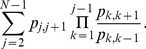
(8)The signature of the formation of a metastable mixed equilibrium is that the expected time to fixation becomes extremely large, so much that actually observing fixation in a normal realization of the process is very unlikely. We have checked that is what happens (with fixation times of the mutant strategy higher than 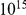) in those invasion processes for which realizations lead to a mixed equilibrium. Therefore the invasion processes cannot be realistically studied by fixation probabilities because mixed equilibria would be completely missed. In any event, and for the sake of completeness, we will compare the results that come from the study of the fixation probabilities with those which come from simulations. This will clearly show the actual importance of allowing mixed states in the different games.

### Acceleration of simulations

The invasion process with the above described discrete dynamics has been studied by simulations. Every invasion process amounted to replace 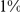 of the resident population by individuals with the invading strategy. For every resident population and any invading strategy we have carried out 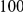 realizations of the process. If part of these realizations ended up in a certain equilibrium, a link was added to the graph going from the node corresponding to the resident population to the node corresponding to the new equilibrium. This link is weighted with the fraction of the 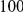 realizations that led to it.

If implemented like that, the process becomes very slow when the probability of selecting from the population two different individuals is very small. In order to solve this problem, we have introduced an accelerated process which avoids all steps in which two identical individual are selected and thus the process remains frozen. To that end, let us note that the probability that in a given step of the Markov process an individual using strategy 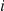 is replaced by another with strategy 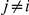 if there are 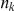 individuals using strategy 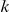 in the population (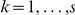, with 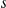 the number of different strategies) is, according to (6),
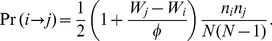
(9)Therefore there will be a change in the composition of the population with probability
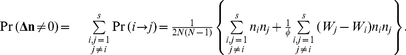
(10)Here 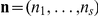 denotes the population vector and 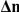 its change after one time step. But
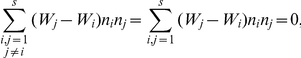
and since 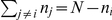,
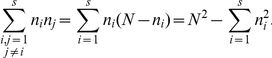
Therefore
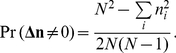
(11)This, together with Eq. (9), yield the probability that 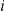 is replaced by 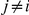 in one time step conditioned on there being a change in the composition of the population, namely
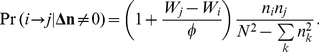
(12)


Using this new process directly is not practical, but we can replace it by a simpler one if we decompose
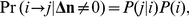
(13)where
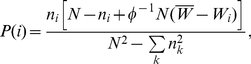
(14)

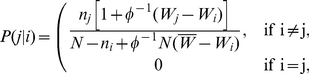
(15)denoting 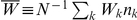. It is straightforward to check that
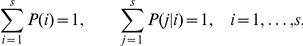
so that 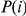 and 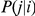 are genuine probabilities. The advantage of this trick is that the accelerated process can be implemented by selecting individuals with strategy 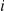 according to the probability distribution 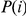 and replacing their strategy with 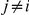 according to 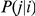.

The original process can be recovered from the accelerated one if between every two contiguous steps of the latter we insert an exponential process with probability 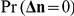 (complementary to (11)). The life expectancy of this process is
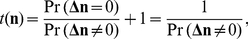
(16)which is finite as long as the population is heterogeneous.

Thus, given a realization of the accelerated process in which the sequence of compositions is 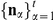, we can estimate the average of a given observable 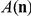 as
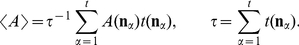
(17)


Simulations are run until the average composition 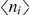 remains constant (plus or minus 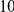 individuals) for an average time 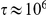.

### Markov chain for the invasion graph

The resulting invasion graph for a given pair of values 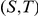 has an associated Markov chain. If 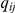 denotes the fraction of invasions that lead the resident community of node 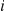 to the new community 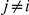 and 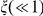 denotes the invasion (or mutation) rate, then matrix 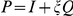, where the diagonal of 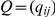 is defined so that 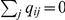, is the transition probability matrix of the Markov chain associated to the invasion graph.

From the theory of Markov chains we know that a permutation of indexes yields the form [41]

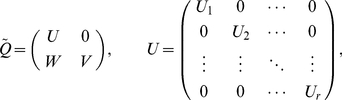
(18)for matrix 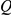. The set of nodes (denoted 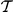) corresponding to sub-matrix 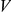 are transient nodes; the remaining ones are recurrent nodes. The latter are subsequently divided into independent disjoint sets, each of which is formed by the nodes involved in each one of the matrices 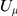, 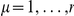 (denote with 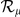 the corresponding set).

The probability 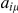 that the Markov chain ultimately enters the set 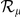 if it started off from node 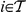 is determined as
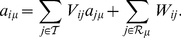
(19)Defining matrices 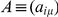 and 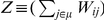, this equation can be written as
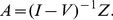
(20)The value
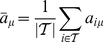
(21)is the average of these probabilities over all transient nodes.

On the other hand, if the Markov chain ends up in set 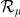, the asymptotic probability distribution it reaches on the nodes of 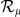 is given by the vector 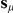, determined as 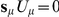.

## Results

### Quasi-stationary invasion process

**Figure 1:**
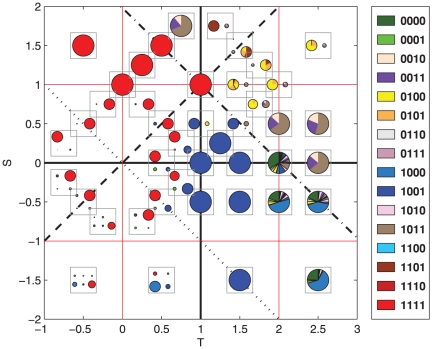
Composition of the recurrent sets for each studied game. The centers of the squares correspond to the pairs 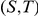 that define those games. Each strategy is assigned a different color (color code in the separate column on the right). Within each square there may be one or more pie charts. Each of them represents a different recurrent set. The sizes of the pies are proportional to the average probabilities of reaching them from the transient states (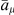). Pie sectors separated with thick lines correspond to different nodes of the recurrent set. Their sizes are proportional to their probabilities in the stationary state (given by the components of the vectors 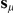). If a sector is of one color it means that the node corresponds a pure strategy; if it is subdivided in smaller sectors with different colors it means that the node corresponds to a mixed strategy, the different colors representing the coexisting strategies. The sizes of these sub-sectors are proportional to their fraction within the mixed strategy.

Following the construction of our model and the corresponding approach to find all its quasi-stationary configurations (as discussed in ‘Methods’) we have studied by simulation the whole parameter space of symmetric 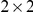 games as given by representative points for different regions. For each pair of values for the payoffs 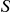 and 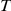, we will describe the asymptotic behavior of the population by giving the probabilities with which the different nodes of the recurrent set 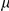 are visited, 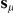, as well as the probabilities (if there is more than one recurrent set) 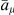 to reach each of them. These data are summarized in a pictorial way in Fig. 1. For those readers interested in the quantitative composition of the equilibria and detailed listing of all the strategies present, we have collected all the information as tables, one for each one of the four quadrants of the 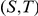 plane, available in Tables 1–4.

**Table 1:** Recurrent sets found in the Snowdrift game quadrant.

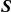	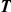	strategies in recurrent set	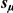	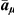
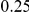	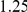	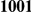	1	1
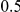	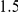	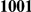	1	0.70
		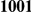	1	0.30
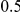	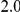	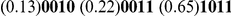	1	1
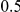	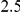	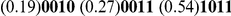	1	1
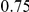	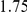	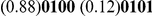	1	0.57
		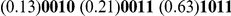	1	0.43
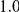	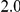	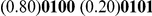	1	0.69
		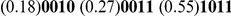	1	0.31
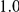	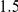	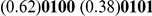	0.58	0.73
		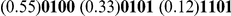	0.40	
		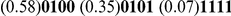	0.02	
		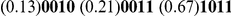	1	0.27
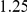	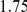	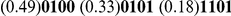	1	0.80
		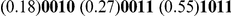	1	0.20
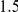	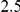	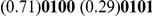	0.99	0.74
		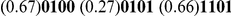	0.01	
		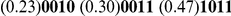	1	0.26
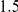	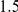	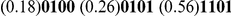	1	0.72
		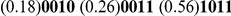	1	0.22
		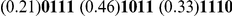	1	0.06
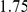	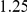	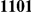	1	0.73
		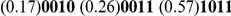	1	0.27

Recurrent sets found for every pair 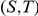 in the Snowdrift game quadrant (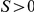, 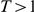). Different boxes for the same pair of 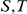 values denote different recurrent sets. The four digits denote the strategies present in the corresponding set. The notation 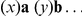 denotes mixed equilibria in which strategies 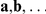 enter with fractions 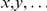 respectively. For each strategy the probability to find it in the recurrent set is given in column 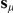. Also listed is the average probability with which the different recurrent sets are reached (column 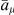).

**Table 2 pone-0035135-t002:** Recurrent sets found in the Harmony game quadrant.

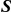	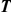	strategies in recurrent set	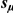	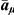
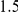	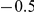	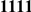	1	1
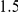	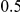	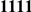	1	1
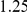	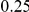	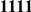	1	1
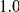	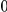	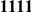	1	1
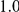	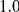	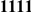	1	1
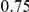	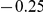	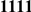	1	0.91
		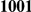	1	0.09
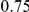	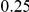	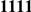	1	0.88
		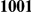	1	0.12
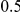	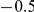	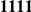	1	0.86
		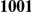	1	0.09
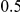	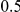	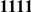	1	0.78
		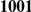	1	0.22
		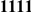	1	0.85
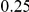	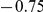	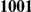	1	0.12
		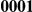	1	0.03
		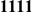	1	0.54
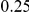	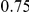	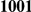	1	0.37
		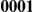	1	0.09
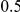	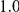	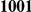	1	0.73
		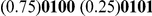	1	0.27
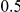	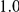	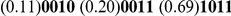	1	1

Same as Table 1 for the Harmony game quadrant (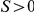, 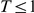).

**Table 3 pone-0035135-t003:** Recurrent sets found in the Prisoner's Dilemma game quadrant.

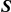	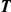	strategies in recurrent set	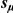	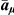
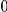	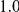	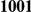	1	1
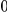	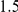	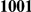	1	1
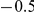	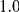	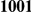	1	1
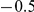	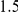	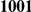	1	1
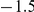	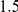	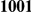	1	1
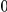	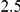	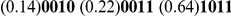	1	1
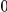	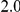	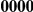	0.34	1
		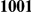	0.10	
		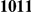	0.10	
		*all others except* 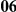	0.46	
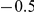	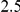	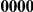	0.19	1
		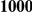	0.43	
		*all others except* 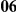	0.43	
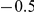	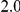	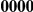	0.20	1
		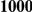	0.49	
		*all others except* 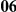	0.31	
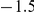	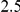	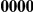	0.24	1
		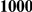	0.62	
		*all*	0.14	

Same as Table 1 for the Prisoner's Dilemma game quadrant (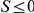, 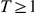).

**Table 4:** Recurrent sets found in the Stag Hunt game quadrant.

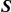	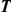	strategies in recurrent set	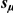	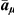
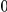	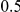	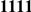	1	0.60
		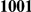	1	0.22
		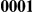	1	0.18
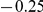	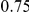	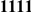	1	0.40
		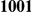	1	0.46
		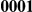	1	0.14
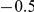	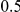	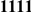	1	0.55
		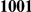	1	0.32
		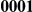	1	0.13
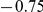	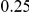	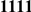	1	0.64
		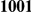	1	0.16
		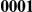	1	0.12
		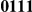	1	0.08
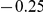	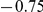	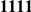	1	0.66
		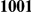	1	0.14
		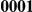	1	0.06
		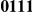	1	0.14
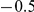	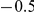	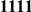	1	0.66
		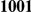	1	0.14
		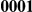	1	0.07
		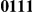	1	0.13
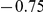	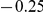	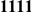	1	0.54
		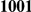	1	0.11
		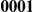	1	0.07
		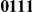	1	0.13
		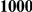	1	0.15
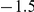	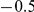	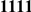	1	0.41
		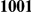	1	0.10
		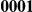	1	0.10
		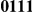	1	0.08
		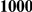	1	0.24
		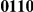	1	0.07
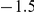	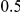	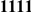	1	0.17
		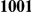	1	0.21
		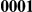	1	0.09
		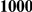	1	0.53

Same as Table 1 for the Stag Hunt game quadrant (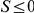, 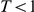).

Figure 1 represents the asymptotic states of the invasion process with pie charts. There is one such diagram for every recurrent set of the Markov chain of the invasion process, and their respective sizes are proportional to the probabilities of ending up in each of them. If a recurrent set is made of different states, each one is assigned a sector of the pie, with an angle proportional to the stationary probability of the Markov chain. Colors code for strategies. If a state corresponds to a homogeneous population (hence a true stationary state of an invasion process) then its sector will have the color that corresponds to its strategy. If the state is a mixed equilibrium (hence a quasi-stationary, or metastable, state of an invasion process) then the sector is subdivided in colored sub-sectors, each of a size proportional to the population share that the corresponding strategy has.

**Figure 2:**
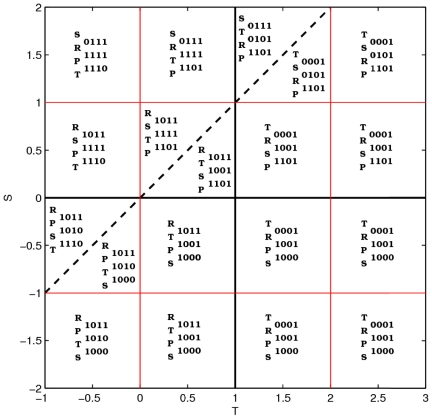
Distribution of the payoffs and the different win-stay, lose-shift strategies. Strategies are ordered according to decreasing aspiration level [32] in the different regions of the 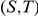 plane.

At first glance, Fig. 1 seems very complicated, but after a more careful study patterns begin to appear, corresponding to the different types of games and a few additional, well defined parameter regions that we will introduce below. To interpret it and understand the origin of the results, it is important to identify the four main classes of games: beginning with the upper left corner and proceeding in clockwise order, the four quadrants (we will call them quadrants even if the 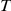 axis is centered around 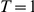 and not 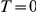) correspond to Harmony, Snowdrift, Prisoner's Dilemma and Stag Hunt. On the other hand, previous works pointed out the importance of the WSLS strategies [31], [32]. These strategies are defined in the following way: an individual who follows a WSLS strategy opts by the same action that carried out in the previous round if and only if she obtained a payoff higher than her aspiration level. Since there are four possible payoffs, there are three possible aspiration levels (disregarding aspirations levels higher than the highest or lower than the lowest possible payoffs). A consequence of the changes in the ordering of payoffs in different regions of the diagram is that the strategies belonging to the WSLS category change along the diagram. In order to make it easier for the reader to follow our discussion, we have included an additional plot, Fig. 2, where we summarize the payoffs and WSLS strategies sorted by aspiration level for every parameter region we are studying. Following [32], we will call *ambitious* the WSLS strategy that is only happy with the highest possible payoff, *balanced* the one that is content with the two highest ones, and *modest* the strategy that only intends to avoid the lowest possible payoff. As an example, for the typical region of the Prisoner's Dilemma (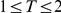, 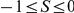) 0001 is ambitious, 1001 is balanced (this is the original Pavlov strategy as identified in [31]) and 1000 is modest.

A striking feature of Fig. 1 worth mentioning is the existence of games with more than one recurrent set. The meaning of this is that the end state for these games is contingent on the initial state of the population and the particular history of invasions that has occurred. The relative probabilities of reaching one or another of these recurrent sets reflect the number of histories that end up in each of them. But no matter how small this probability is, once the population reaches one of these end states, it is uninvadable.

Let us begin the presentation of our results by the region 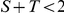 of the 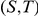 plane, i.e., the part of the diagram below the dot-dashed line in Fig. 1. Generally speaking, except for very high temptation values in the Prisoner's Dilemma quadrant, in this region we only find recurrent sets consisting of a single type of strategy (except for the game with 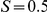, 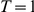, at the border between Harmony and Snowdrift, for which there is a second small recurrent set where 0100 and 0101 coexist in a mixed strategy). In agreement with previous research, for the strict Prisoner's Dilemma game with not so large temptation values, the unique absorbing node turns out to be the balanced WSLS strategy (Pavlov, 1001). An example of this result can be seen for the parameters 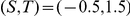, where a blue large dot represents the absorbing set formed only by Pavlov. The same happens for the 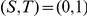 point, but starting from there and as one enters further in the Harmony game quadrant, there is a smooth transition in which an additional absorbing set appears, corresponding to the strategy AllC (1111), the balanced WSLS in this region above the 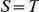 line (dashed in Fig. 1; see also Fig. 2). In fact, above that line the equilibrium configuration is practically always AllC, with a residual presence of Pavlov in a few points. Therefore, our first result can be phrased by saying that balanced WSLS strategies represent the equilibrium configurations of the Prisoner's Dilemma (for not so large temptations, specifically 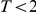) and the Harmony games.

The above conclusion applies in general to the region comprised between 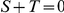 (dotted line in Fig. 1) and 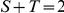, but things are not so simple when one looks at the part of this region that belongs to the Stag Hunt. The Stag Hunt quadrant is abundant in games with history-contingent end states. This is not surprising because in Stag Hunt coordinating pays, and coordination can be achieved by several different strategies–among them AllC (1111) and Pavlov (1001), the two strategies that are exchanging the role of balanced WSLS. However we also see evidence for 0001 becoming a new absorbing state, albeit in a smaller –but noticeable–proportion than AllC and Pavlov. The reason why the strategy 0001 appears in the Stag Hunt quadrant is because it does coordinate with itself (like the other two), although in this case it oscillates every step between CC and DD. The presence of this strategy supports the conclusions of [42], where it was pointed out that WSLS strategies are not efficient in some of these games (we will see below that this is also the case in the remaining region of the Snowdrift game).

**Figure 3:**
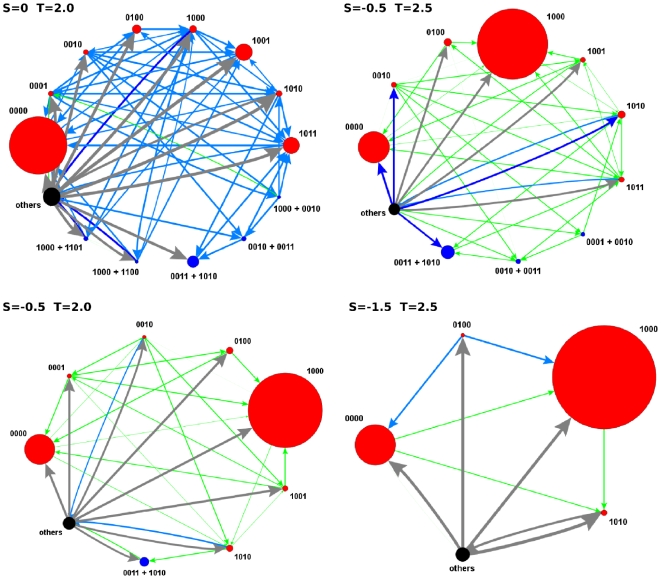
Graphs of the recurrent set in the 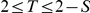
 **games.** Vertices show the most representative (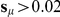) pure (red) and mixed (blue) states. The rest of states are grouped in a single vertex (black). The size of a vertex is proportional to its corresponding value of 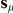. Arrows show the transition probabilities, with widths and colors proportional to their values normalized to the minimum probability (in ascending order: green, light blue, dark blue and grey).

Let us now move to other regions of the 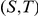 plane; in particular, let us consider the parameter set limited by 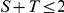 and 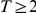, which belongs to the Prisoner's Dilemma. Here we find that the successful strategy is not Pavlov anymore but, instead, equilibria are almost ergodic on the whole set of strategies. In fact, Pavlov becomes a secondary strategy in so far as trajectories within the recurrent set do not spend much time in it. The two most important strategies in the recurrent set are 1000, the modest WSLS one, and AllD; interestingly, 1000 becomes dominant–in the sense that the population spends more time using it–as 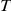 increases and 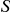 decreases. It is worth noting that this modest WSLS strategy can be identified with GRIM, the strategy that cooperates until the partner defects, and then turns to defection forever. On the other hand, the fact that nearly all strategies appear in the equilibria has important implications: it means that almost every strategy can invade and be invaded by some of the others. This is clearly seen in Fig. 3, where we represent the invasion subgraphs of the recurrent sets of the four games in this region. Focusing as an example in the 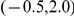 game, that corresponds to Axelrod's tournaments [12], we confirm that there is not a unique dominant strategy and, in addition, we see that the evolution of the system is quite complicated. Note in particular that transitions between AllD and GRIM are almost never direct, but rather they proceed through intermediate populations; thus, 0000 becomes destabilized and evolves towards 0010 or 1010 (the famous TFT), to proceed from there to GRIM through Pavlov. Similar cycles are repeated in the other three games, as can be seen in Fig. 3. An interesting observation that also arises from these plots is that as 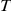 increases and 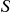 decreases, not only GRIM becomes more important, but in addition the structure of the invasion graph simplifies largely, and direct transitions from AllD to GRIM become possible, the cycle being completed through TFT and subsequently to others to go back to GRIM. Therefore, we see that in the large temptation region Pavlov is not the dominant strategy anymore, and this role is now played, to different extents, by GRIM and AllD.

The reader may be puzzled by the fact that the scenario we are depicting for the 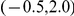 game is so different from the outcome of Axelrod's tournaments [12]. One should bear in mind though that our dynamics and Axelrod's is very different. Axelrod confronted every pair of strategies, which interacted along 200 rounds and accumulated the payoffs obtained through the tournament. TFT was the best scoring strategy. But this is very different from the dynamics we are exploring here, where every new strategy attempts to invade a resident, stable population. This difference was already noted by Nowak and Sigmund [19] in their simulations confronting random mixtures of different strategies.

There are still two parameter regions left: the Stag Hunt quadrant below the 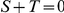 line and the portion of the Snowdrift quadrant with 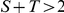. As already noted above, these two sets cannot be explained in terms of WSLS strategies, at least not entirely; in addition, the structure of the equilibria is different in the two games. For the Stag Hunt game, we always find several absorbing states. This multi-stability is the signature of this game, even when played one shot. The ultimate reason is the existence of two Nash equilibria in the one-shot game, which gives players some freedom in devising different coordinating strategies. The relative importance of the different absorbing states–measured in terms of the probability of reaching them–depends on the parameter values, but in general the most relevant strategy, i.e., the one appearing more often as a result of the evolution, is AllC. As we go down the quadrant, Pavlov, initially significant, loses much of its importance and its probability as a possible evolutionary stable population decreases with respect to GRIM–which also plays here the role of a modest WSLS strategy. As a matter of fact, for very negative 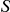 and 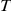 close to (but below) 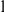, GRIM substitutes even AllC as the most relevant strategy.

As for the part of the diagram with 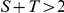, practically in all cases we find mixed equilibria, and more often than not two different mixed equilibria form disjoint recurrent sets. The key to understand these results is to note that in this region, any payoff which is a combination of 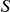 and 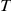 is higher than the highest fixed payoff (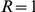). Since an individual and her co-player have to opt for a different action (cooperate or defect) to get 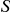 or 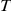 payoffs, they need to anti-coordinate their actions, so they can benefit alternatively in even or odd rounds. Hence, the best strategies are two mixed states: 0010+0011+1011 and 0100+0101(+1101). Indeed, an individual that plays one of the strategy from the last group continues cooperating after obtaining 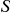 and defecting after 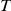. In this manner, one of the player is always obtaining 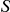 and the other 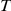. If one of them mistakes her action (which will eventually occur), the combination of two strategies forces them back to the 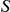-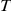 dynamics in one or two rounds. The same process takes place for the first mixed state, but in this case both players switch actions all the time, i.e., when one obtains 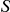 she changes to defection and when she gets 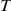 she goes back to cooperation. It is interesting to note that as S becomes the largest payoff, the strategy 1101 becomes more important (see upper left corner of the Snowdrift quadrant in Fig. 1). This strategy is the modest WSLS one in the region, and is highly cooperative as well, defecting only if needed to anti-coordinate with the opponent. Therefore, even if the description of the successful strategies in this region is in general not compatible with WSLS strategies, they still play a role in their modest version.

### Fixation probabilities

As discussed in ‘Methods’, in a finite population the dynamics eventually leads to fixation of one of the strategies–either the resident or the invader. If the population is large, the presence of quasi-stationary mixed states may increase the fixation time exponentially with the size of the population. If the population is large, fixation will never be observed in practice; however, in small populations and with very small mutation (invasion) rates the fixation process becomes meaningful. In order to make a complete discussion of this problem we will address here the description of the invasion process in terms of fixation probabilities [43]. A plus of this analysis is that it will reveal the relevance of the mixed states in the final outcome of the process.

**Figure 4:**
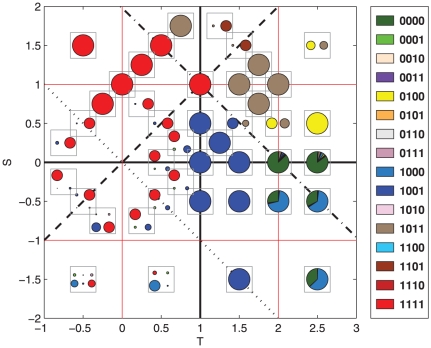
Composition of the recurrent sets for each studied game. The plot is the same as Figure 1, but measuring the transition probabilities as probabilities of absorption.

We represent in Fig. 4 the results of the Markov chain constructed out of fixations and fixation probabilities, using the same conventions as in Fig. 1. Given two strategies, a resident one and an invader, fixation of either one has always a nonzero probability to occur, even though most often one of them is very small. For the sake of simplicity fixation probabilities smaller than 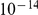 has been neglected in this plot.

The first obvious difference between Figs. 1 and 4 is that all mixed equilibria have disappeared in the latter. This is very important for the region 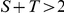 (in the Snowdrift quadrant). In general, Fig. 4 is simpler than Fig. 1, but remarkably the main general features remain. Thus, the stripe delimited by 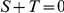 and 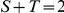 shows again the smooth transition from Pavlov in the Prisoner's Dilemma with not so large temptations to AllC in the Harmony game, both being the balanced WSLS strategies in their corresponding parameter sets. In the region where we found the mixed equilibria before, the Snowdrift game with 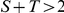, we now have only three strategies: 0100, 1011 and 1101. The last one, the modest WSLS option, is only relevant for larger 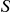, whereas the other two are strategies that tend to anti-coordinate with the opponent, changing action with a probability of 50%.

Another noticeable difference is that sometimes the simulations show a few more absorbing states than the fixation analysis (e.g., for 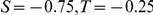). We have checked that this is a result of insufficient statistics in simulations. Indeed, some states appear as absorbing because the probability that they are invaded by other strategy is always smaller than the inverse of the number of realizations (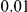 in our case). We have checked that by increasing precision in simulations these absorbing states disappear and the two scenarios approach each other. Anyway the average probabilities of being absorbed in these fake absorbing states are always small (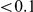) so they hardly arise as the final result of the evolution.

Another difference between the two schemes is that the probabilities of the absorbing nodes and recurrent sets are slightly different in the 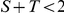 region. This is not just due to insufficient statistic but a deeper effect. As it turns out, mixed states do influence the asymptotic probability distribution of the recurrent sets even when they do not belong to them, because the probability that a mixed state arises or is invaded by one or another strategy affects in turn the probabilities of the invaded or invading strategies. This fact is even clearer for the games with 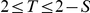, where the mixed states are part of the recurrent sets.

All in all, we can conclude that the analysis using fixation probabilities does not change qualitatively our main conclusions, and only affects the existence of the mixed states, which are replaced by a few of the participants in the mixed states found in the simulations.

## Discussion

In this paper, we have carried out a thorough study of the evolutionary successful strategies in iterated symmetric 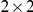 games, focusing on quasi-deterministic memory-one strategies. To this end, we introduced an approach that has proven very fruitful in ecosystem assembly studies [34]–[36], whose basic idea is to study the process as isolated invasions which lead from a population composition to another one with some probability, and to build a Markov chain out of these invasion transitions. We have implemented two versions of the procedure, one involving an individual based model, that allows to identify mixed equilibria with extremely long absorbing times–hence relevant to actual observations–and another more standard one based on fixation probabilities, that leads to the truly asymptotic results. The agreement between both approaches is very good and provides a test of our findings as well as a check on the validity range of the simulation approach. We have also introduced an accelerated version of the dynamics so that the simulations can be performed in an affordable computing time. This acceleration is quite general and valid for many other birth-death processes.

The main picture that arises from our study highlights the importance of WSLS strategies, as first found in [31]. Our results confirm that, generally speaking, balanced WSLS (those aspiring to intermediate payoffs) strategies are the successful ones in the Prisoner's Dilemma and in the Harmony game. On a larger scale, some type of WSLS strategy, be it balanced or modest (aspiring to the second lowest payoff) is always relevant to understand the equilibrium structure of the games. Thus, GRIM, a modest WSLS strategy, is important in the Prisoner's Dilemma for large temptations, whereas 1101 appears with large probability in the Snowdrift game. As for the structure of the selected populations, we have found that when 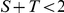, absorbing states consisting of only one strategy appear, whereas above that line and to the right of 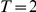 both, complex recurrent states or mixed states, result from the evolution of the population. The latter is the region where the risk in cooperating (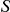) and the temptation to defect (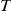) are very large, and their influence on the evolution of the population is considerably larger than that of the other two payoffs. Therefore, it pays to have a strategy focusing in obtaining these two payoffs, which leads to anti-coordination in the Snowdrift game and to invadability of almost any strategy in the Prisoner's Dilemma for large temptations, when anti-coordinating is very detrimental for the player choosing the lower payoff action. For this last case, we have been able to build the detailed invasion graphs and to completely characterize how strategies replace each other, the complexity of the recurrent state decreasing with increasing absolute values of 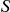 and 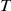.

A final remark is in order concerning the lessons from our study. We have found that ambitious WSLS strategies are never successful for any of the games we have studied. This is likely to occur because such strategies need a population to exploit, as the players using them need to benefit from their opponent's good will in order to obtain the largest payoff. In so doing, they lead the exploited strategies to extinction, and subsequently cause their own disappearance as they become invadable by more modest WSLS strategists, that fare well against each other. On the other hand, our results point out to the importance of identifying the kind of social dilemma one is involved in and, in particular, to realize that one is in a Snowdrift type of situation, because in this case and unless 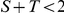, a WSLS approach will not succeed. The challenge for the players is then to try to take turns in choosing the most beneficial actions and be so generous as to avoid insisting on being always the player with the largest payoff. Such an ambitious version of anti-coordination is indeed possible for some time when there is coexistence in the population of exploiter and exploited individuals, but in the end only alternating anti-coordination can prevail. Once again, extreme ambition does not pay, a conclusion that is confirmed in the large temptation regime of the Prisoner's Dilemma by noticing the very important role played by GRIM in keeping AllD at bay, preventing it from becoming fixed in the population. Therefore, we see that modest ambitions regarding the payoffs are the rule of thumb to deal successfully with these 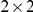 social dilemmas.

## References

[1] Hammerstein P (2003). Genetic and Cultural Evolution of Cooperation (Dahlem Workshop Report 90).

[2] Maynard Smith J, Szathmary E (1995). The Major Transitions in Evolution.

[3] Darwin C (1871). The Descent of Man, and Selection in Relation to Sex.

[4] Hamilton WD (1964). The genetical evolution of social behaviour I.. J Theor Biol.

[5] Hamilton WD (1964). The genetical evolution of social behaviour II.. J Theor Biol.

[6] Trivers RL (1971). The evolution of reciprocal altruism.. Q Rev Biol.

[7] Nowak MA (2006). Five rules for the evolution of cooperation.. Science.

[8] Dugatkin LA (1997). Cooperation among animals: An evolutionary perspective.

[9] Hammerstein P, Hammerstein P (2003). Why is reciprocity so rare in social animals? a protestant appeal.. Genetic and Cultural Evolution of Cooperation (Dahlem Workshop Report 90).

[10] Kappeler PM, van Schaik CP (2006). Cooperation in Primates and Humans: Mechanisms and Evolution.

[11] Hublin JJ (2009). The prehistory of compassion.. Proc Natl Acad Sci USA.

[12] Axelrod R (1984). The Evolution of Cooperation.

[13] Lordkipanidze D, Vekua A, Ferring R, Rightmire GP, Agustí J (2005). The earliest toothless hominin skull.. Nature.

[14] Gracia A, Arsuaga JL, Martínez I, Lorenzo C, Carretero JM (2009). Craniosynostosis in the Middle Pleistocene human Cranium 14 from the Sima de los Huesos, Atapuerca, Spain.. Proc Natl Acad Sci USA.

[15] Rand DG, Ohtsuki H, Nowak MA (2009). Direct reciprocity with costly punishment: Generous tit-for-tat prevails.. J Theor Biol.

[16] Axelrod R, Hamilton WD (1981). The evolution of cooperation.. Science.

[17] Selten R, Hammerstein P (1984). Gaps in harley's argument on evolutionarily stable learning rules and in the logic of tit for tat.. Behav Brain Sci.

[18] Nowak MA, Sigmund K (1989). Oscillations in the evolution of reciprocity.. J Theor Biol.

[19] Nowak MA, Sigmund K (1992). Tit for tat in heterogeneous populations.. Nature.

[20] Nowak MA, Sigmund K (1993). A strategy of win-stay, lose-shift that outperforms tit-for-tat in the prisoner's dilemma game.. Nature.

[21] Kraines D, Kraines V (1989). Pavlov and the Prisoner's Dilemma.. Theory and Decision.

[22] Fudenberg D, Maskin E (1990). Evolution and cooperation in noisy repeated games.. Am Econ Rev.

[23] Imhof LA, Fudenberg D, Nowak MA (2005). Evolutionary cycles of cooperation and defection.. Proc Natl Acad Sci USA.

[24] Imhof LA, Fudenberg D, Nowak MA (2007). Tit-for-tat or win-stay, lose-shift?. J Theor Biol.

[25] Fudenberg D, Rand DG, Dreber A (2012). Slow to anger and fast to forgive: Cooperation in an uncertain world.. Am Econ Rev.

[26] Rapoport A, Guyer M (1966). A taxonomy of 2×2 games.. General Systems.

[27] Sigmund K (2010). The Calculus of Selfishness.

[28] Nowak MA, May RM (1992). Evolutionary games and spatial chaos.. Nature.

[29] Rubinstein A (1986). Finite automata play the repeated Prisoner's Dilemma.. J Econ Theory.

[30] Binmore KG, Samuelson L (1992). Evolutionary stability in repeated games played by finite automata.. J Econ Theory.

[31] Nowak MA, Sigmund K (1993). Chaos and the evolution of cooperation.. Proc Natl Acad Sci USA.

[32] Nowak MA, Sigmund K, El-Sedy E (1995). Automata, repeated games and noise.. J Math Biol.

[33] Sugden R (1986). The economics of rights, co-operation and welfare.

[34] Capitán JA, Cuesta JA, Bascompte J (2009). Statistical mechanics of ecosystem assembly.. Phys Rev Lett.

[35] Capitán JA, Cuesta JA (2011). Species assembly in model ecosystems, I: Analysis of the population model and the invasion dynamics.. J Theor Biol.

[36] Capitán JA, Cuesta JA, Bascompte J (2011). Species assembly in model ecosystems, II: Results of the assembly process.. J Theor Biol.

[37] Kollock P (1998). Social dilemmas: the anatomy of cooperation.. Annu Rev Sociol.

[38] Roca CP, Cuesta J, Sánchez A (2009). Evolutionary game theory: temporal and spatial effects beyond replicator dynamics.. Phys Life Rev.

[39] Hofbauer J, Sigmund K (1998). Evolutionary Games and Population Dynamics.

[40] Seneta E (2006). Non-negative Matrices and Markov Chains.

[41] Karlin S, Taylor HM (1975). A First Course in Stochastic Processes.

[42] Posch M, Pichler A, Sigmund K (1999). The efficiency of adapting aspiration levels.. Proc R Soc Lond B.

[43] Fudenberg D, Imhof LA (2006). Imitation processes with small mutations.. J Econ Theory.

